# Metformin inhibits the proliferation of hepatocellular carcinoma cells through inducing ferroptosis analyzed by phosphoproteomics

**DOI:** 10.3389/fonc.2025.1531420

**Published:** 2025-05-30

**Authors:** Jihan Sun, Xi Yu, Yuying Shan, Manyun Dai, Andrea Gaucherot, Caide Lu, Shuqi Mao

**Affiliations:** ^1^ Department of Hepatopancreatobiliary Surgery, The Affiliated Lihuili Hospital, Ningbo University, Ningbo, Zhejiang, China; ^2^ French Blood Establishment (EFS), National Institute of Health and Medical Research (INSERM), Joint Research Unit Regulation of Immunity for therapeutic innovation in Graft, Host, Tumoral and inflammatory associated diseases (UMR RIGHT) F-25000, Franche-Comté University, Besançon, France; ^3^ School of Public Health, Ningbo University Health Science Center, Ningbo, China; ^4^ Laboratory of the Metabolic Adaptations to Exercise Training Under Physiological and Pathological Conditions (AME2P), Clermont Auvergne University, Clermont-Ferrand, France

**Keywords:** metformin, hepatocellular carcinoma, ferroptosis, phosphoproteomics, cell viability

## Abstract

**Introduction:**

This study aims to investigate the effects of Metformin on hepatocellular carcinoma cell lines, cell lines and explores the molecular mechanisms underlying.

**Methods:**

Bel-7402 and HepG2 cells were treated with varying concentrations of Metformin and ferrostatin-1 to assess cytotoxicity using the MTT assay. Protein expression and phosphorylation changes were analyzed through a phosphoproteomics approach and further bioinformatics analysis, including Gene Ontology (GO) and Kyoto Encyclopedia of Genes and Genomes (KEGG) databases. The data from phosphoproteomics were confirmed by western blot. Metformin treatment significantly reduced cell viability in a concentration- and time-dependent manner. Phosphoproteomics analysis identified differentially expressed proteins (DEPs) primarily associated with ferroptosis and cellular metabolism. Further GO and KEGG analyses revealed the involvement of DEPs in nucleotide biosynthesis, membrane transport, and metabolic pathways. Then the bioinformatic data were verified through MTT and western blot.

**Results and discussion:**

The results showed that ferrostatin-1 could partly reverse the inhibitory effects of Metformin, suggesting the involvement of ferroptosis. Moreover, the modulation of autophagy and ferroptosis-related proteins by Metformin was confirmed by western blot. Our findings demonstrate that Metformin induces ferroptosis in Bel-7402 and HepG2 cell lines, potentially offering a novel therapeutic strategy for the treatment of hepatocellular carcinoma. The phosphoproteomics analysis provides insights into the molecular mechanisms by which Metformin exerts its anti-cancer effects.

## 1 Introduction

Hepatocellular carcinoma (HCC) accounts for the majority of primary liver cancer and represents the fourth leading cause of worldwide cancer deaths (1, 2). It commonly develops through a multistep process from liver cirrhosis to low-grade dysplastic nodule, high-grade dysplastic nodule, early HCC and progressed HCC (3). Ferroptosis, a novel type of cell death due to iron-dependent phospholipid peroxidation, has received extensive attention for its lethal effect on tumor cells (4, 5). The dysregulation of ferroptosis is highly associated with the progression of HCC, and the induction of ferroptosis is also proposed as a potential promising strategy for HCC therapy (6). Accordingly, it is pertinent to elucidate the mechanisms of ferroptosis in HCC to eventually identify useful therapeutic approaches for this disease.

Metformin, a biguanide commonly used as a first-line agent for type 2 diabetes, has attracted increasing attention for its anti-cancer properties, particularly in liver-related malignancies (7, 8). Beyond its glucose-lowering activity via AMPK activation, metformin has been reported to suppress tumor progression through multiple mechanisms, including cell cycle arrest, inhibition of epithelial–mesenchymal transition (EMT), and modulation of metabolic stress (9, 10). Recent studies also demonstrate that metformin can trigger ferroptosis in HCC cells via pathways involving ATF4/STAT3 signaling or suppression of the p62–Keap1–Nrf2 antioxidant axis, thereby enhancing sensitivity to chemotherapeutic agents such as sorafenib (11, 12, (13). Additionally, ferroptosis sensitivity has been shown to be tightly regulated by the p53–SLC7A11–GPX4 axis in liver cancer cells, offering new insight into how metformin might influence this cascade (14). Notably, recent studies have highlighted the cell-line–dependent heterogeneity in ferroptosis sensitivity among HCC models. For instance, Hu et al. reported that gene expression and antioxidant capacity related to ferroptosis varied significantly between HCC cell lines, underscoring the need for parallel experimental validation across multiple models (15).

Importantly, metformin’s effects appear to intersect with autophagy and redox regulation, yet the phosphoproteomic landscape underlying these effects in HCC remains poorly defined. Protein phosphorylation plays a key role in regulating cellular signaling and is tightly linked to stress responses, metabolism, and cell death pathways (16, 17). Dysregulation of phosphorylation-mediated processes often participates in the initiation and progression of many human diseases, including cancers (18). Phosphoproteomics has evolved as a powerful tool to reveal phosphorylation sites of kinases and can provide important insights into kinases that can be targeted for therapeutic intervention; in particular, the phosphoproteomics-based approaches have been widely used to investigate cancer cells and develop personalized treatment (19, 20).

In this study, we hypothesize that metformin inhibits HCC cell proliferation by inducing ferroptosis through phosphorylation-mediated signaling networks. To test this hypothesis, we employed integrated phosphoproteomic profiling, bioinformatic pathway analysis, and *in vitro* validation using ferroptosis inhibitors. Our aim is to delineate the signaling events by which metformin exerts its anti-HCC effects, with the ultimate goal of identifying actionable ferroptosis-associated regulatory nodes.

## 2 Materials and methods

### 2.1 Human cell cultures

The Bel-7402 and HepG2 cell lines, both widely used for hepatocellular carcinoma research, were acquired from the American Type Culture Collection (ATCC) and cultured under sterile conditions to ensure cell viability and purity. The cells were maintained in a culture flask with Dulbecco’s Modified Eagle’s Medium (DMEM, Gibco, Cat. No. 11-965-092, USA), supplemented with 10% fetal bovine serum (FBS, Gibco, Cat. No.11560636, USA) and 1% penicillin-streptomycin (ThermoFicher Scientific, Cat. No.15323671, USA). The cultures were maintained in a humidified incubator at 37°C with 5% CO2. Passaging of the cells was carried out when the cells reached approximately 80-90% confluence, 0.25% Trypsin-EDTA (ThermoFisher Scientific, Cat. No. 25200072) was used to digest cells, the cell suspension was then transferred to new culture flasks for further experiments.

### 2.2 MTT assay of metformin and ferrosatin-1 treatment

To assess the cytotoxic effects of Metformin and Ferrostatin-1 on Bel-7402 and HepG2 cells, MTT (3-(4,5-dimethylthiazol-2-yl)-2,5-diphenyltetrazolium bromide) assay was employed to determine the cell viability and proliferation. Briefly, a total of 10000 cells in 200 μL medium were pipetted into 96-well plates to grow for 24h. They were then pre-treated with 1 µM Ferrostatin-1 for 24 hours (21) before exposure to Metformin (Santa-Cruz, Cat. No. 1115-70-4) (0, 1, 2, 5, 10 and 20mM, diluted in phosphate buffer saline) for 24, 48, or 72 hours. After treatment, 20 μL of MTT reagent was added to each well, and the cells were incubated for 4 hours at 37°C. Then the medium was discarded, and formazan precipitate was dissolved in 150 μL DMSO. Absorbance at 570 nm was measured by a plate reader (Tecan Spark^®^, Männedorf, Switzerland). Each experimental group was conducted in triplicate.

### 2.3 Protein extraction and preparation

Total cellular proteins were extracted using RIPA buffer (ThermoFisher Scientific, Cat. No. 89900), supplemented with a cocktail of protease and phosphatase inhibitors (Roche) to prevent degradation and dephosphorylation of target proteins. Extracts from each sample were reduced with 2mM DTT for 1 hour at 56°C, and subsequently alkylated with sufficient iodoacetamide for 1 hour at room temperature in the dark. Then 4 times the volume of precooled acetone was mixed with samples by well vortexing and incubated at -20°C for at least 2 hours. Samples were then centrifuged and the precipitation was collected. After washing twice with cold acetone, the pellet was dissolved by dissolution buffer, which contained 0.1 M triethylammonium bicarbonate (TEAB, pH 8.5) and 6 M urea. Protein concentration was determined again by Bradford protein assay.

### 2.4 Peptide digestion and phosphopeptide enrichment

Digestion of 5 mg of protein was performed with Trypsin Gold at a 1:50 enzyme-to-substrate ratio for 16 hours at 37°C. Peptides were desalted using a C18 cartridge and dried by vacuum centrifugation. For phosphopeptide enrichment, peptides were redissolved in 250 mM acetic acid with 30% acetonitrile, adjusted to pH 2.5-3.0, and processed using phos-select iron affinity gel according to the manufacturer’s protocol. The bounded peptides were eluted, dried and desalted using peptide desalting spin columns (Thermo Fisher, 89852).

### 2.5 LC-MS/MS analysis and data interpretation

Peptides were analyzed using an EASY-nLC™ 1200 UHPLC system (Thermo Fisher) coupled with an Orbitrap Q Exactive HF-X mass spectrometer (Thermo Fisher) operated in data-dependent acquisition (DDA) mode and positive polarity. Full MS scans were acquired at a resolution of 60,000 (at m/z 200) over a scan range of 350–1,500 m/z. The automatic gain control (AGC) target for MS1 was set to 3 × 10^6^, with a maximum injection time of 50 ms. The top 20 most intense precursor ions were selected for higher-energy collisional dissociation (HCD) with a normalized collision energy (NCE) of 27%. MS/MS scans were acquired at a resolution of 15,000 (at m/z 200), using an isolation window of 1.6 m/z, an AGC target of 1 × 10^5^, and a maximum injection time of 45 ms. Dynamic exclusion was enabled with a duration of 30 seconds to avoid repeated selection of precursor ions.

The resulting spectra were searched against a protein database using Proteome Discoverer 2.2 (PD 2.2, Thermo), with carbamidomethylation as a fixed modification, and oxidation of methionine, phosphorylation of serine (S), threonine (T), and tyrosine (Y), and N-terminal acetylation as variable modifications. Protein identification was performed at a false discovery rate (FDR) < 1.0%, and label-free quantification was applied. The protein quantitation results were statistically analyzed by the Mann–Whitney test. Differentially expressed proteins (DEPs) were defined based on fold-change (ratio > 4 or < 0.25) and statistical significance (*P* < 0.05).

Functional analysis of proteins and DEPs was conducted using Gene Ontology (GO), InterPro (IPR), COG (Clusters of Orthologous Groups), and KEGG (Kyoto Encyclopedia of Genes and Genomes) databases. GO and IPR analysis was conducted using the InterProScan-5 program against the non-redundant protein database (including Pfam, PRINTS, ProDom, SMART, ProSiteProfiles, and PANTHER). STRING-db (http://string.embl.de/) was used to predict potential protein–protein interactions based on related species. Motif enrichment analysis was conducted using the motif-x algorithm, focusing on a 7-amino acid window surrounding each phosphosite (occurrence > 20, *P* < 10^−6^), and visualized with WebLogo. Phosphorylation site–kinase relationships were predicted using the NetPhorest algorithm.

### 2.6 Western blot

Total cellular proteins were extracted using RIPA buffer (ThermoFisher Scientific, Cat. No. 89900), supplemented with a cocktail of protease and phosphatase inhibitors (Roche, Cat. No. 04693132001) to prevent degradation and dephosphorylation of target proteins. Protein concentrations were quantified using the BCA Protein Assay Kit (ThermoFisher Scientific, Cat. No. A55860), ensuring accurate loading of samples. 50 μg of each protein extract was resolved by SDS-PAGE and electrophoretically transferred onto a nitrocellulose membrane. The membranes were then incubated with primary antibodies specific for ATG9 (Abcam, ab108338, diluted 1:1000), ATG5 (Abcam, ab108327, diluted 1:1000), ATG16 (Abcam, ab188642, diluted 1:1000), P62 (Abcam, ab109012, diluted 1:10000), Beclin-1 (Abcam, ab207612, diluted 1:2000), Ic3b (Abcam, ab231078, diluted 1:1000), SLC7A (Abcam, ab236669, diluted 1:500), GPX4 (Abcam, ab125066, diluted 1:1000), ASCL4 (Abcam, ab155282, diluted 1:10000), and β-actin (Abcam, ab8227, diluted 1:1000) overnight at 4°C to ensure efficient antigen-antibody binding. After thorough washing with Tris-buffered saline with Tween (TBST) to remove unbound primary antibodies, the membranes were incubated with an HRP-conjugated secondary antibody (Bio-Rad, diluted 1:2000) for 1 hour at room temperature to amplify the signal. To visualize the immunoreactive bands, the membranes were developed using a chemiluminescent substrate (ThermoFisher Scientific, Cat. No. 34080). The luminescent signal was detected by ChemiDoc™ Imaging System (Bio-Rad) and relative expression levels normalized to β-actin were calculated

## 3 Results

### 3.1 Cellular viability response of Bel-7402 and HepG2 treated by metformin

**Figure 1:**
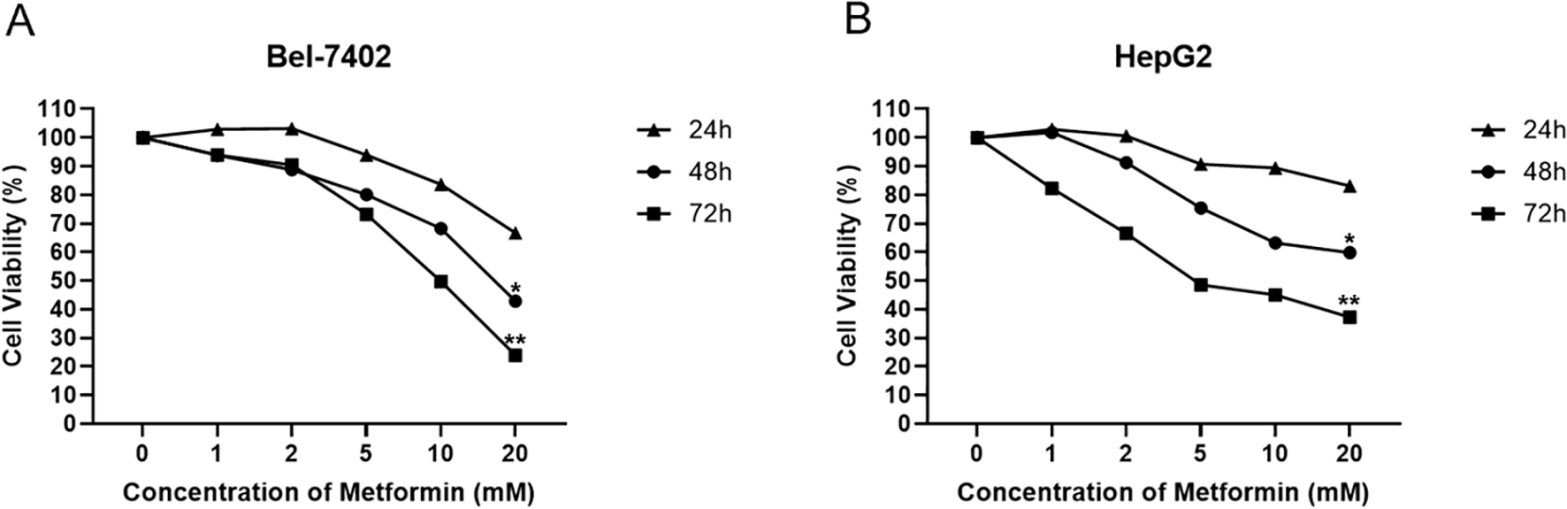
Cellular viability response of Bel-7402 **(A)** and HepG2 **(B)** treated by Metformin with different concentrations at 24, 48 and 72 hours using MMT assay. Data are presented as mean ± SD (n = 3). Statistical analysis was performed using one-way ANOVA followed by Tukey’s *post hoc* test. *P*< 0.05 (*) and *P*< 0.01 (**) indicate statistically significant differences compared to the 0 mM (control) group.

The cell viability of two cell lines including Bel-7402 and HepG2 treated with Metformin of multiple concentrations was detected by MTT assay. The results showed that the cell viability of Bel-7402 cells indicated a negative correlation with Metformin concentration at different time points of 24, 48 and 72 hours. Among these, the Bel-7402 cells treated with 20mM Metformin at 72 hours exhibited the lowest cell viability compared with other groups, thus this concentration was selected for further experiment. Similarly, the results in HepG2 cells also represented the higher concentration of Metformin or longer duration of treatment, the lower cell viability of cells (**Figure 1**). These results indicated the inhibitory effect of Metformin on the proliferation of Bel-7402 and HepG2.

### 3.2 Phosphoproteomics of Bel-7402 and HepG2 treated by metformin

**Figure 2:**
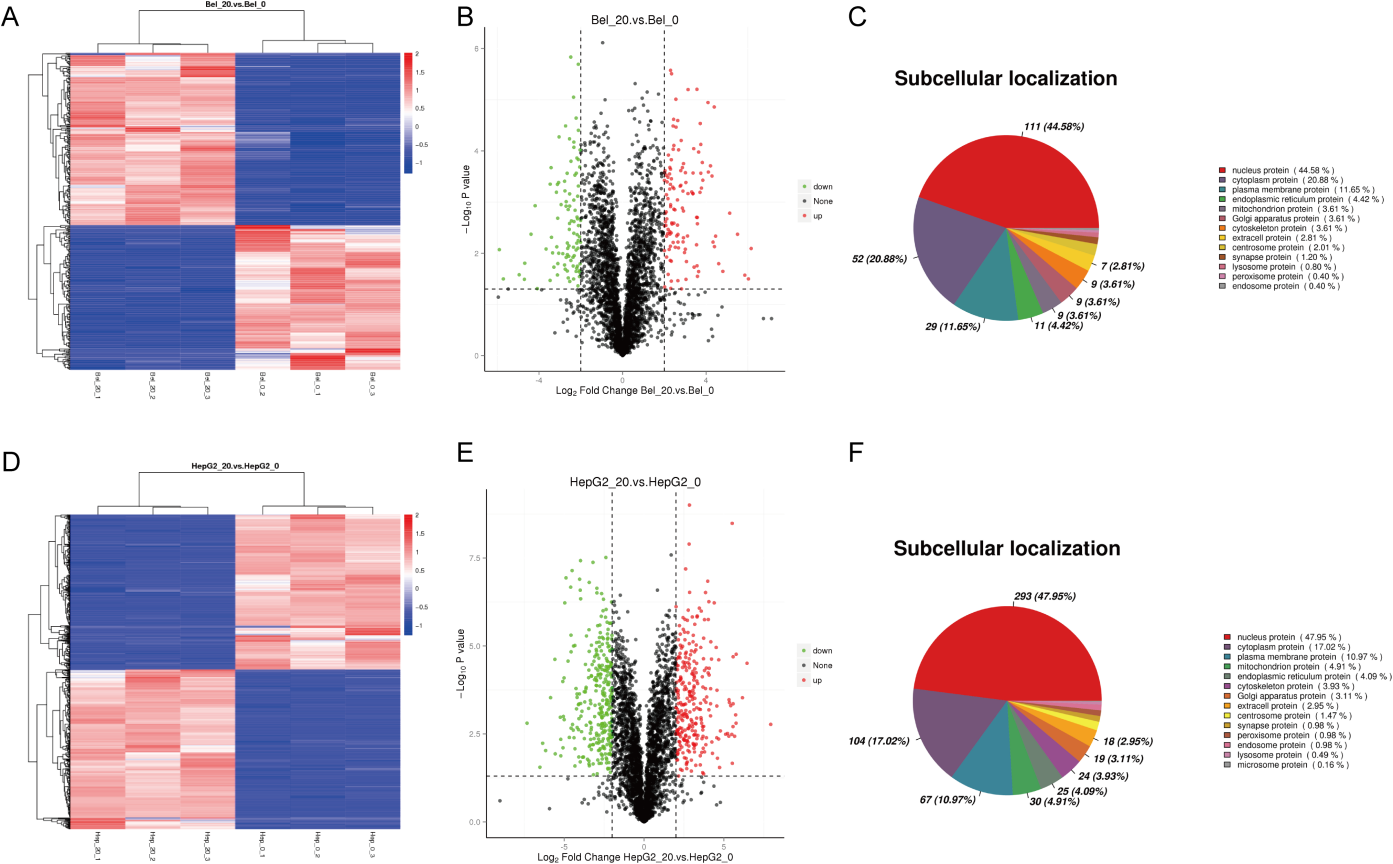
Phosphoproteomics of Bel-7402 and HepG2 treated by Metformin. The heatmap, volcano plot and subcellular localization analysis in Bel-7402 **(A–C)** and HepG2 **(D–F)** cell lines. Significantly altered phosphoproteins (n = 132 for Bel-7402; n = 148 for HepG2) were identified using p < 0.05 and fold-change >4 or <0.25 as cutoffs. Red and blue indicate upregulation and downregulation, respectively.

To further explore the molecular regulation mechanism of Metformin in hepatocellular carcinoma cells, phosphoproteomics and bioinformatics analysis were performed. As shown in **Figure 2**, the heatmap and volcano plot showed a distinct difference between the control group (Bel_0/Hep_0) and Metformin-treated groups (Bel_20/Hep_20) either in Bel-7402 or HepG2 cells. Furthermore, subcellular localization analysis revealed that the alternation of DEPs was mainly located in the nucleus, with 44.58% in Bel-7402 and 47.95% in HepG2 cells, followed by cytoplasm proteins.

### 3.3 Gene ontology and KEGG analysis of phosphoproteomics

**Figure 3:**
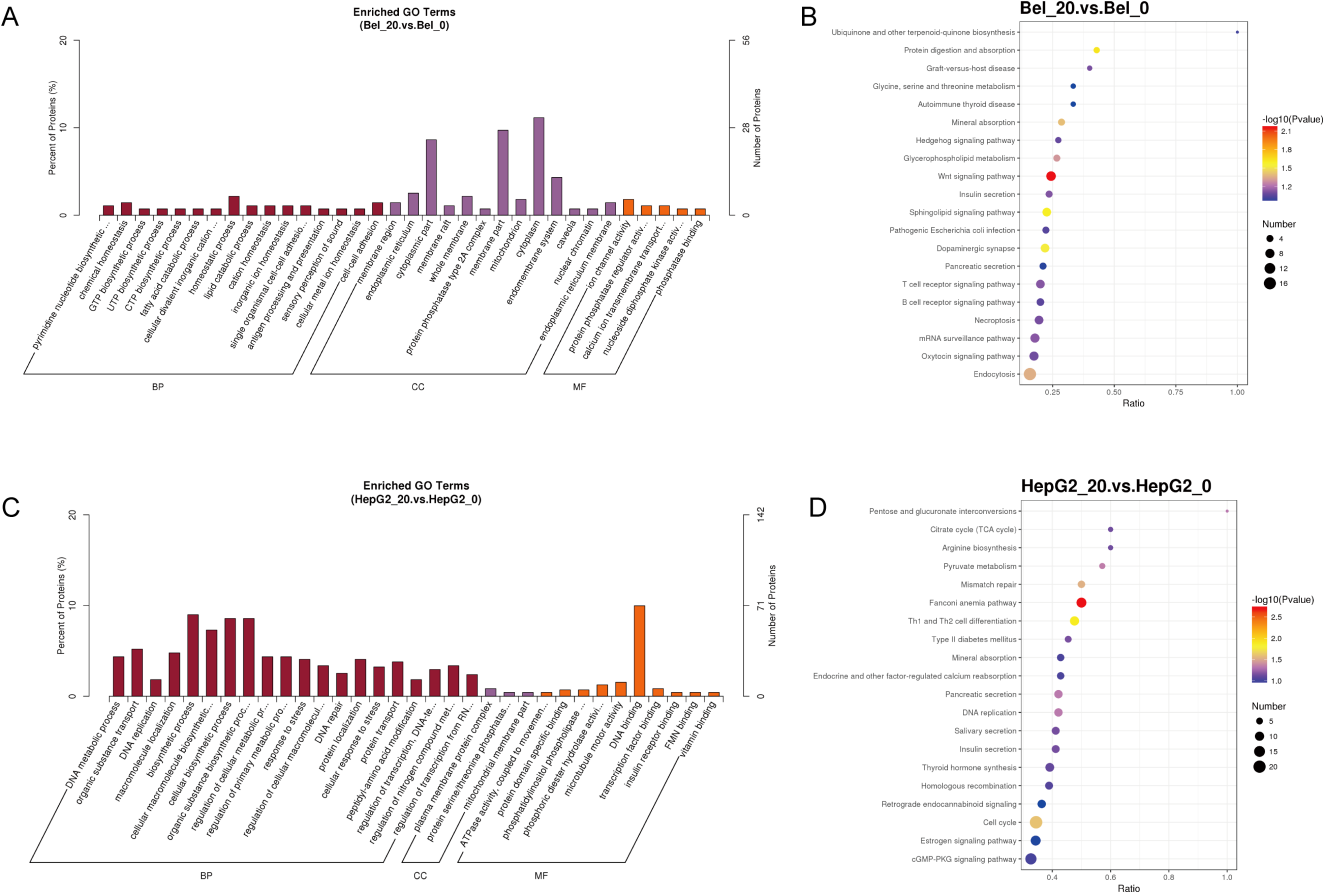
Gene ontology analysis of phosphoproteomics. The Enriched GO Terms and KEGG pathway analyses in Bel-7402 **(A, B)** and HepG2 **(C, D)** cell lines.

The function of DEPs between control and Metformin-treated groups in two cell groups was further analyzed by Gene Ontology analysis. Specifically, the enriched GO analysis showed that the top 5 terms in Bel-7402 cells were pyrimidine nucleotide biosynthetic process, chemical homeostasis, GTP biosynthetic process, UTP biosynthetic process and CTP biosynthetic process in Biological Process (BP); membrane region, endoplasmic reticulum, cytoplasmic part, membrane raft, whole membrane in Cellular Component (CC); and ion channel activity, protein phosphatase regulator activity, calcium ion transmembrane transporter activity, nucleoside diphosphate kinase activity, phosphatase binding in Molecular Function (MF) (**Figure 3A**). The KEGG pathway analysis of Bel-7402 cells suggested the significant difference in the pathway between Bel_0 and Bel_20 was mainly focused on the biosynthesis and metabolism regulation, such as Ubiquinone and other terpenoid-quinone biosynthesis, Glycine, serine and threonine metabolism, and Insulin secretion, etc (**Figure 3B**).

As for HepG2 cells, the top 5 enriched terms analyzed by GO were DNA metabolic process, organic substance transport, DNA replication, macromolecule localization and biosynthetic process in BP; plasma membrane protein complex, protein serine/threonine phosphatase complex, mitochondrial membrane part, clathrin coat of trans-Golgi network vesicle, clathrin coat of coated pit in CC; and ATPase activity, protein domain specific binding, phosphatidylinositol phospholipase C activity, phosphoric diester hydrolase activity and microtubule motor activity in MF (**Figure 3C**). Moreover, the KEGG pathway analysis of HepG2 cells suggested Pentose and glucuronate interconversions, Citrate cycle (TCA cycle), Arginine biosynthesis, Pyruvate metabolism and mismatch repair, suggested a primary alternation of biosynthesis and metabolism regulation in HepG2_0 and HepG2_20, similarly to the GO data in Bel-7402 cells (**Figure 3D**).

### 3.4 Ferroptosis pathway analysis based on KEGG in two cell lines

Notably, KEGG analysis revealed that differentially expressed protein enriched in ferroptosis pathway, as well. Furthermore, several studies shown that ferroptosis is closely interconnected with various molecular pathways and cellular processes, including immunological signaling, transcription factors, other cell death modalities, and metabolic pathways, which were all mentioned in our phosphoproteomics results above (**Figure 3**). This complex network of interactions highlights that ferroptosis is a key molecular process involved in the Metformin inhibitory effect on hepatocellular carcinoma (22–24).

**Figure 4:**
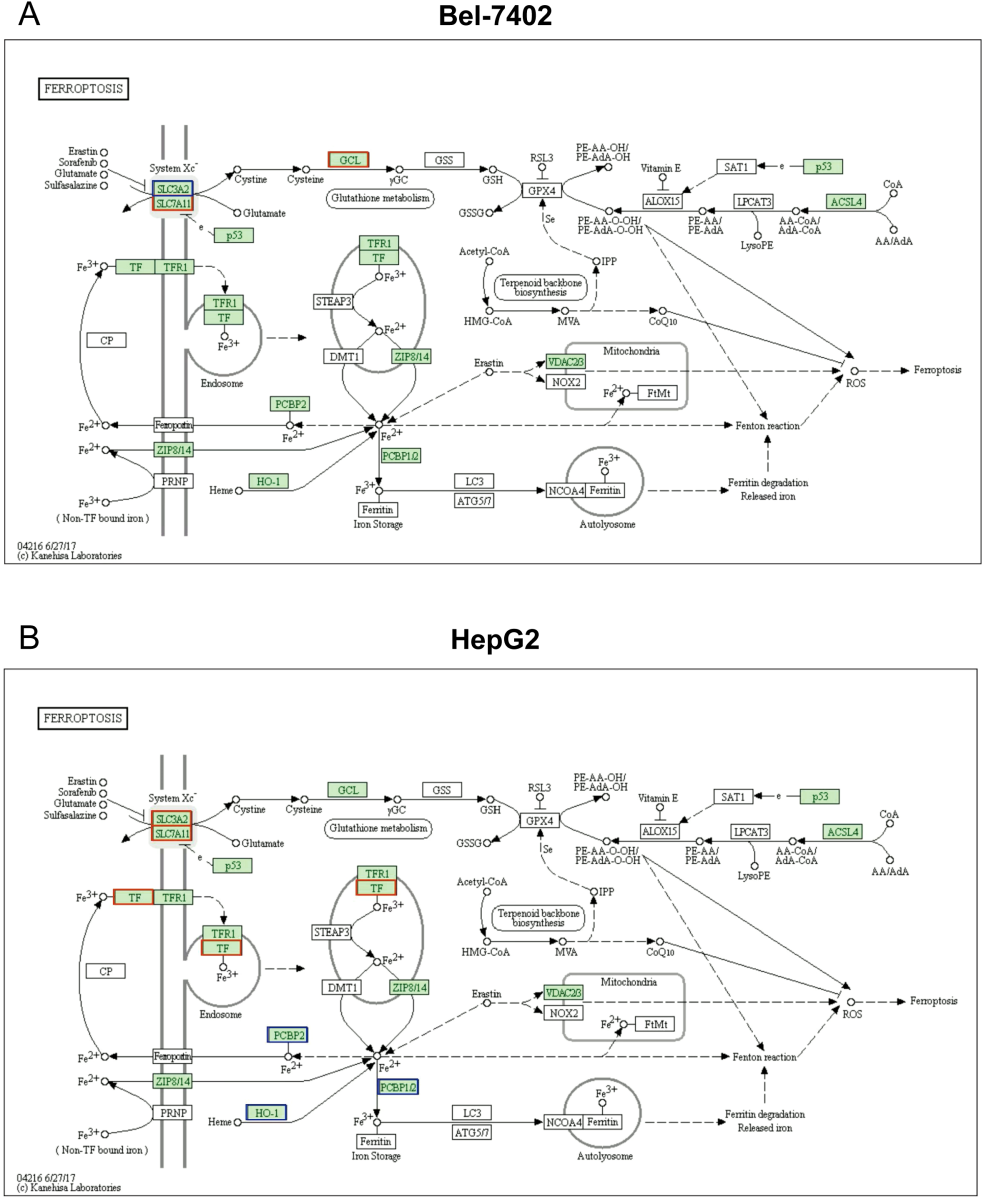
Ferroptosis pathway analysis based on KEGG in Bel-7402 **(A)** and HepG2 **(B)** cell lines.

As represented in **Figure 4**, the downregulation of SLC3A2, and the upregulations of SLC7A11 and GCL signals were found to participate in the ferroptosis process, these proteins were also found to be a significant difference between Bel_0 and Bel_20 groups. Consistently, several DEPs related to ferroptosis were also determined in HepG2_0 and HepG2_20, such as the overexpression of SLC3A2, SLC7A11, TF, and downregulation of PCBP2 and HO-1.

### 3.5 The role of ferroptosis in metformin-mediated inhibition of hepatocellular carcinoma

**Figure 5:**
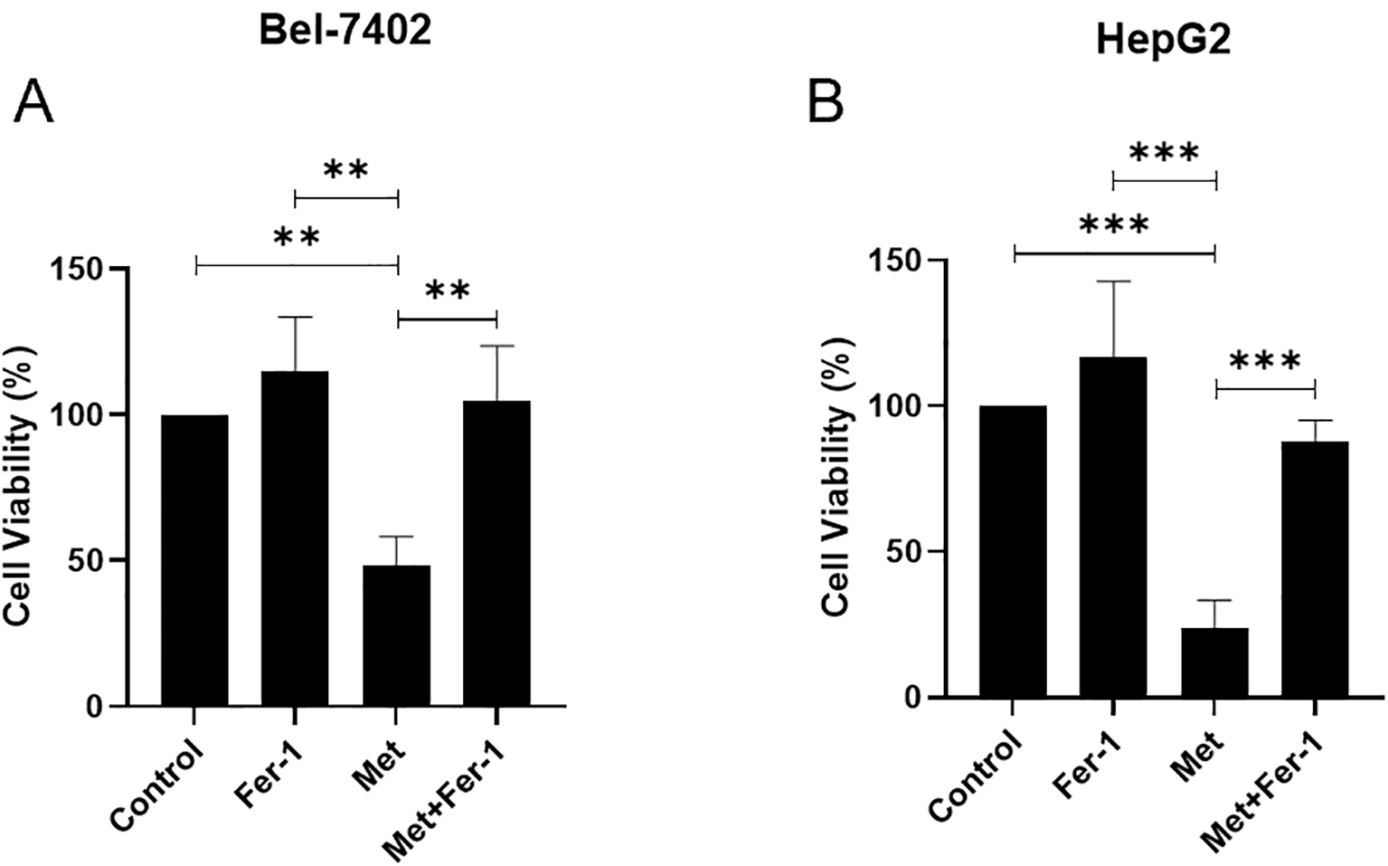
The cell viability is affected by Metformin through ferroptosis in Bel-7402 **(A)** and HepG2 **(B)** cell lines. Fer-1, ferrostatin-1; Met, Metformin. P<0.01(**), P<0.001 (***).

MTT assay demonstrated that metformin could significantly suppress the cell viability of Bel-7402, while its inhibitory role could be partly reversed by ferrostatin-1 (all p<0.05) (**Figure 5A**), also known as Fer-1, a potent and selective inhibitor of ferroptosis. In line with Bel-7402 cells, there is a dramatic difference between Fer-1 and Metformin treatment groups in HepG2 cells (p<0.05), and the suppressive impact of Metformin could be partly eliminated by Fer-1 (p<0.05), as shown in **Figure 5B**.

### 3.6 The ferroptosis and autophagy-related protein expressions affected by metformin

**Figure 6:**
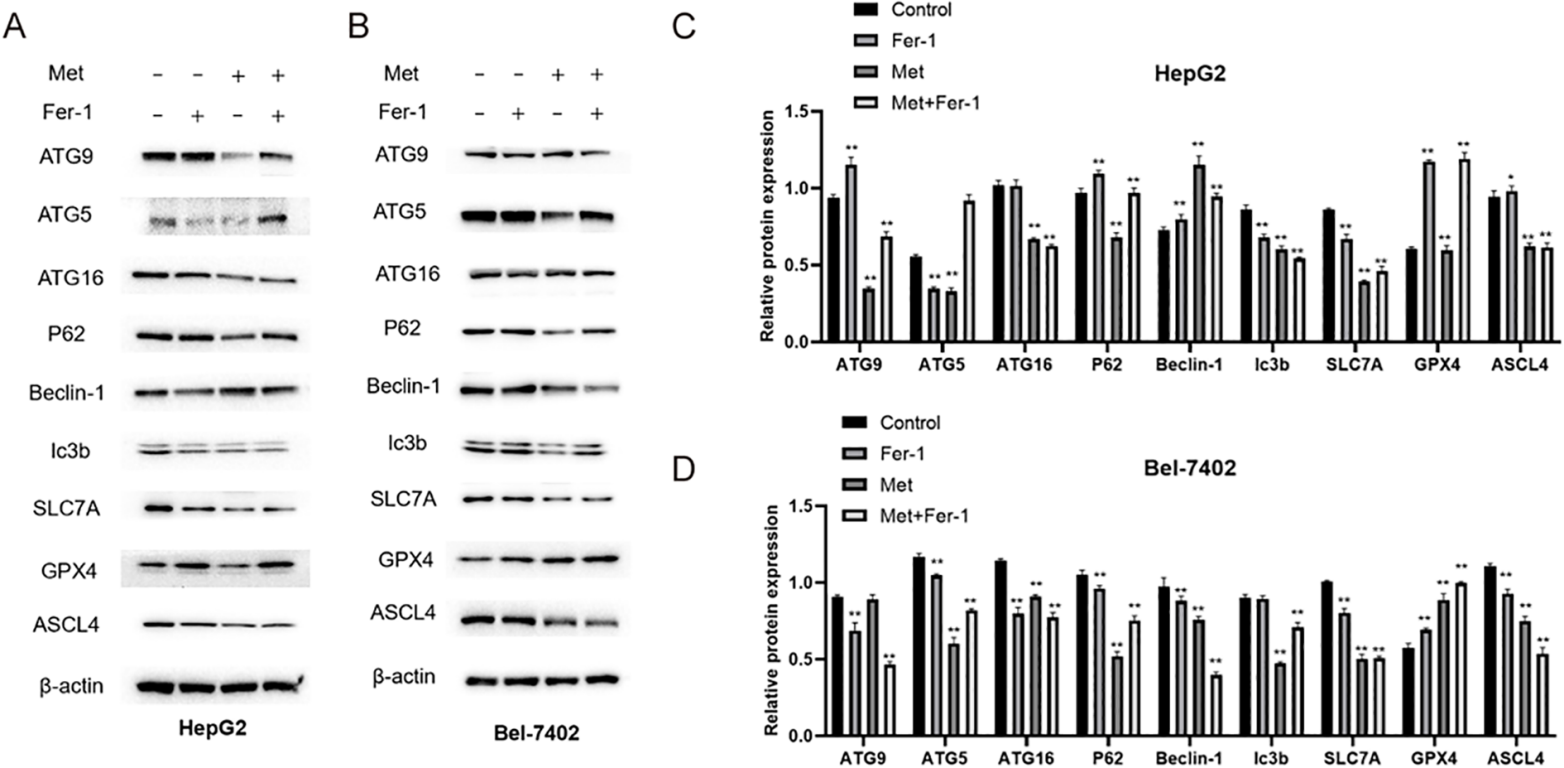
The protein expressions of ferroptosis and Autophagy-related protein detected by WB in HepG2 **(A, C)** and Bel-7402 **(B, D)** cell lines. Fer-1, ferrostatin-1; Met, Metformin. Statistical analysis was performed using one-way ANOVA followed by Tukey’s *post hoc* test. *P*< 0.05 (*) and *P*< 0.01 (**) indicate statistically significant differences compared to the 0 mM (control) group.

Based on the partially reversed MTT results and the fact that ferroptosis closely interacts with autophagy (24), WB was performed to detect the ferroptosis and Autophagy-related protein expressions in HepG2 and Bel-7402 cells. The expression levels in HepG2 were shown in **Figures 6A, C**, the data showed that the Autophagy-related proteins including ATG9, ATG5, P62 were decreased in Metformin group, and could be recused by Fer-1, while ATG16 and Ic3b did not show an obvious rescue effect after Fer-1 intervention in Metformin-treated HepG2 cells. The ferroptosis proteins such as SLC7A and GPX4 showed a significant inhibition after Metformin treatment, and could be upregulated after Fer-1 intervention. No significant change was noticed in ASCL4 between Metformin and Metformin+Fer-1 groups.

As for Bel-7402 cell line, its expression results were shown in **Figures 6B, D**, the protein expressions of ATG5, P62, Ic3b were downregulated in Metformin group, and were rescued by Fer-1 treatment. However, ATG9 and ATG16 showed opposite results that the Metformin could elevate their expression whereas this effect could be partly reversed by Fer-1 intervention. Intriguingly, ferroptosis-relevant proteins including SLC7A, GPX4 and ASCL4 showed different tendencies in Bel-7402 cells, indicating an intrinsic interaction of the ferroptosis process.

## 4 Discussion

Phosphoproteomics is a powerful approach for the identification and quantitation of phosphorylation sites at the system level, paving the way toward deeper understanding of the regulatory mechanisms underlying cancers (25, 26). Importantly, the current study employed phosphoproteomics approach in combination with bioinformatics analysis and *in vitro* studies and revealed the underlying mechanisms by which metformin acts in HCC. Metformin treatment decelerated the proliferation of HCC cells via ferroptosis induction, consequently suppressing the progression of HCC.

Several studies have shown that metformin acts as a therapeutic drug for the treatment of HCC as it can regulate biological functions of cancer cells, including proliferation, migration, invasion, angiogenesis, colony formation, apoptosis, and autophagy (27–29). Here, in this study, we adopted metformin treatment for *in vitro* assay and demonstrated that metformin could dramatically impede the proliferation in HCC cells in a dose- and time-dependent manner. This finding is in agreement with a previous study which shows that HCC cells treated with metformin exhibit significantly reduced cell proliferative, migratory and invasive capacities, during which alternative splicing of LGR4 plays a central role (30). Another study has documented that metformin counteracts HCC cell proliferation markedly via increasing cells arrested at the G2/M phase, dependent on the suppressing effect of miR-378 on CDK1 expression (31). Additionally, use of metformin contributes to repression of hepatoma cell proliferation and promotion of cell cycle arrest at the G0/G1 phase in a dose-dependent manner through AMP-activated protein kinase and its upstream kinase LKB1 to elevate p21/Cip1 and p27/Kip1 and decline cyclin D1 (32).

Metformin is believed to exert its anti-cancer effect by both direct cellular and indirect systemic mechanisms of actions; in the direct model, metformin inhibits mitochondrial respiration in cancer cells, downregulates ATP levels, and activates AMPK, triggering inhibition of mTORC1, and ultimately inhibiting cell growth. In the indirect model, metformin enhances insulin sensitivity, and increases the uptake of glucose in the cell, diminishing circulating levels of insulin (an essential factor for cell growth promotion), and thus impairing cell proliferation (12, 33, 34). Notably, subsequent results of this study demonstrated that the ability of metformin to induce ferroptosis may be a mechanism underlying its anti-proliferative effect in HCC cells, based on phosphoproteomics, bioinformatics analysis and *in vitro* studies. In much accordance to our results, a recent study has documented that metformin can augment the anti-proliferative effect of sorafenib on HCC cells HepG2 and Huh-7 through ferroptosis induction by blocking the p62-Keap1-Nrf2/HO1 signaling pathway (11). *In vitro* experiments combined with bioinformatics data vindicated that Fer-1 could partially abolish the inhibitory effects of metformin in HCC. To the best of our knowledge, this study is the first to offer evidence in support of this claim.

As a kind of regulated cell death, ferroptosis is directly triggered through three possible mechanisms: denaturation of relevant proteins on the cellular membrane; damage of the integrity of the cellular membrane; and increase of the permeability of the cellular membrane (35, 36). It is well known that ferroptosis is characterized by iron-dependent lipid peroxidation. The occurrence of lipid peroxidation and accumulation of intracellular iron can result in over-production of reactive oxygen species, which causes cellular damage, ultimately leading to cell death and tumor growth inhibition (37, 38). Indeed, there is increasing evidence that the induction of ferroptosis is a promising therapeutic option for HCC therapy due to its abrogating role in the growth and proliferation of HCC cells (15, 39, 40). GPX4 and SLC7A11 are important ferroptosis markers; downregulation of their expression can sensitize cells to ferroptosis (14, 41). In the present study, SLC7A11 and GPX4 expression showed a significant drop in HCC cells after metformin treatment, while this drop could be annulled by Fer-1 intervention. These results are supported by a previous report by Deng et al. (13). In light of these results, it can be plausible that metformin may be a potential target for ferroptosis-mediated HCC treatment.

Moreover, emerging evidence suggests that metformin may also influence ferroptosis indirectly through the autophagy pathway. Metformin activates AMPK, which inhibits the mTOR pathway and promotes autophagy (42, 43). Autophagy, particularly ferritinophagy—a selective degradation of ferritin—can increase intracellular labile iron levels and promote lipid peroxidation, thereby enhancing ferroptosis (44, 45). In support of this, recent studies in HCC have shown that agents activating AMPK, such as nicotinamide mononucleotide (NMN), induce both autophagy and ferroptosis via phosphorylation-mediated signaling cascades, resulting in tumor suppression (46).

Based on these findings, we propose that metformin may exert its ferroptosis-inducing effect not only through direct modulation of ferroptosis-related proteins such as SLC7A11 and GPX4, but also through autophagy-regulated iron and lipid metabolism pathways. This dual regulatory mechanism may contribute to its anti-proliferative efficacy in HCC. Further studies are needed to validate this hypothesis using autophagy inhibition or AMPK/mTOR modulation models (47–53).

In conclusion, based on phosphoproteomics, bioinformatics analysis and *in vitro* studies, the current research validated the mechanism by which metformin regulated the proliferation of HCC cells. These findings may provide valuable insights into the pathogenesis and therapeutic target of HCC. However, the molecular mechanism of how metformin affects ferroptosis and the resultant cell proliferation in HCC needs to be clarified by further experimental studies. Moreover, the potential of metformin-mediated modulation of autophagy warrants further investigation.

## Data Availability

The original contributions presented in the study are included in the article/**Supplementary Material**. Further inquiries can be directed to the corresponding authors.
