# Supplementary material for: Metformin inhibits the proliferation of hepatocellular carcinoma cells through inducing ferroptosis analyzed by phosphoproteomics
Source: Front Oncol. 2025 May 30;15:1531420. doi: 10.3389/fonc.2025.1531420 (PMC12162293; doi:10.3389/fonc.2025.1531420)
Supplement: Supplementary file 1 [file Table1.docx]

Table S1. Summary of representative pathways and phosphoproteins identified by GO, COG, and KEGG enrichment analysis following metformin (20 mM, 72 h) treatment of HCC cells.

| **Pathway / Category** | **Database** | **Enriched Term / Pathway** | **Representative Phosphoproteins** | **Biological Relevance** |
| --- | --- | --- | --- | --- |
| Cell cycle regulation | GO | Mitotic cell cycle process | CDK1 (pT14/Y15), CCNB1 | Metformin may arrest cells in G2/M phase |
| Ferroptosis | KEGG | Ferroptosis | GPX4 (pS2), ACSL4 (pS447) | Core regulators of iron-dependent cell death |
| Autophagy | KEGG | Autophagy – animal | ATG5 (pS193), ULK1 (pS757) | AMPK/mTOR-linked autophagy pathway |
| AMPK signaling pathway | KEGG | AMPK signaling pathway | PRKAA1 (AMPKα, pT172), Raptor (pS792) | Key metabolic sensor activated by metformin |
| mTOR signaling | KEGG | mTOR signaling pathway | mTOR (pS2448), 4EBP1 (pT37/46) | Downregulated by AMPK, inhibits cell growth |
| Oxidative stress response | GO | Response to oxidative stress | NRF2 (pS40), KEAP1 (pS104) | Redox balance closely linked to ferroptosis |
| Protein processing in ER | KEGG | Protein processing in endoplasmic reticulum | HSPA5 (GRP78, pT516), EIF2AK3 (PERK, pT980) | ER stress may contribute to ferroptosis induction |
| Lipid metabolism | COG | Lipid transport and metabolism | FASN (pS207), SCD1 (pS62) | Lipid remodeling important in ferroptosis |
